# Rare case of postpartum echinococcosis at Chris Hani Baragwanath Hospital

**DOI:** 10.4102/sajid.v40i1.750

**Published:** 2025-10-28

**Authors:** Natasha C. Di Rago, Candice Johnson, Yasmin Adam

**Affiliations:** 1 Department of Obstetrics and Gynaecology, Faculty of Health Sciences, University of the Witwatersrand, Johannesburg, South Africa

**Keywords:** human echinococcosis, parasite, pelvic mass, hydatid disease, uterus, pregnancy, non-endemic area

## Abstract

The authors describe a rare case of uterine infection occurring in the postpartum period, following an otherwise uneventful antenatal and peripartum course in a patient who delivered at 36 weeks of gestation. Originally from rural Eastern Cape with livestock exposure, her case emphasises accurate diagnosis in patients from rural areas presenting with respiratory symptoms, including zoonotic infections as causative organisms.

**Contribution:**

This case underscores the importance of identifying uncommon infections in unusual anatomical sites and atypical settings, enabling early intervention to prevent severe complications.

## Introduction

Human echinococcosis is a zoonotic parasitic infection caused by tapeworms, primarily *Echinococcus granulosus* and *Echinococcus Multillocularis*.^1^ It is endemic in regions where people and livestock live in close contact with dogs, which act as the definitive hosts, while livestock serve as intermediate hosts.^2^ Humans become accidental hosts by ingesting parasite eggs. Once inside the small intestine, adult worms release oncospheres that enter the bloodstream and spread to distant organs, forming cysts.^1^ Although the liver and lungs are the most affected body regions, rare presentations in atypical sites such as the uterus may occur. We present a rare case of uterine hydatid disease in South Africa, associated with pregnancy and subsequent disseminated echinococcosis.

Echinococcosis is caused by six recognised species of *Echinococcus*, but *E. granulosus* and *E. multilocularis* are the primary species infecting humans, causing cystic and alveolar echinococcosis, respectively.^1^ The life cycle of the parasite includes definitive hosts, typically dogs and other canines, which harbour adult tapeworms.^2^ Livestock such as cattle, sheep, goats and pigs act as intermediate hosts carrying the larval form. Humans act as incidental hosts, becoming infected when ingesting parasite eggs from contaminated sources, including water, raw or undercooked vegetables or meat from infected livestock.^3^

Hydatid disease is prevalent in areas where livestock farming is common and animals live in proximity to humans. High prevalence regions include South America, Australia, India, the Middle East, sub-Saharan Africa and the Mediterranean region.^4^ Over 1 million people are affected globally each year.^5^ While national data are limited, a retrospective National Health Laboratory Service (NHLS) study revealed the highest prevalence of echinococcosis in South Africa (excluding KwaZulu-Natal) to be in the Eastern Cape (30.4%) and Northern Cape (18.0%).^6^ The analysis included echinococcus serology, microscopy and histopathology reports from public health institutions.

The clinical course of hydatid disease is variable. Many patients remain asymptomatic for years; cysts may be discovered incidentally on imaging or at autopsy. Clinical presentation is influenced by the size of the cyst, its location and the presence of complications such as rupture, secondary infection or mass effect on surrounding structures.^5^ Although any organ can be affected, over 80% of infections involve the liver and lungs.^3^ Reports of hydatid cysts in atypical locations such as the brain, heart, kidney, soft tissues, breast and uterus are rare.^7^ Uterine involvement is exceptionally rare, with only a few cases reported globally.^7^

## Case presentation

A 19-year-old woman presented to the obstetric unit at Chris Hani Baragwanath Academic Hospital (CHBAH), 22 days after delivering her first child via spontaneous vaginal delivery at 36 weeks of gestation.

Her antenatal care was booked at 20 weeks of gestation, based on her last menstrual period. Care was provided at a primary health clinic in Ennerdale, Johannesburg. She never received a growth ultrasound during her antenatal course. Routine antenatal serology revealed HIV-negative, rhesus-positive and syphilis-negative results. The pregnancy and delivery were uncomplicated. She delivered a 2180 g female neonate with Apgar scores of 8 and 9. Both mother and child were discharged 10 h postpartum in stable condition.

Five months prior to delivery, she had relocated from her hometown of Mthatha in the Eastern Cape to Johannesburg. Her family resided in a traditional rondavel and were involved in livestock farming, particularly cattle husbandry.

She presented with a 5-day history of shortness of breath, dry cough, pleuritic chest pain and night sweats. On clinical assessment, she was hypotensive, tachycardic, tachypnoeic and hypoxic (blood pressure 100/68 mmHg, pulse 113 beats per minute, respiratory rate 25 breaths per min, oxygen saturation 82%, temperature 36.2 °C). Cardiovascular examination was normal; respiratory examination showed decreased air entry in the right middle and lower lung zones. Abdominal palpation revealed a firm, tender pelvic mass measuring 22 cm above the pelvic brim.

**FIGURE 1:**
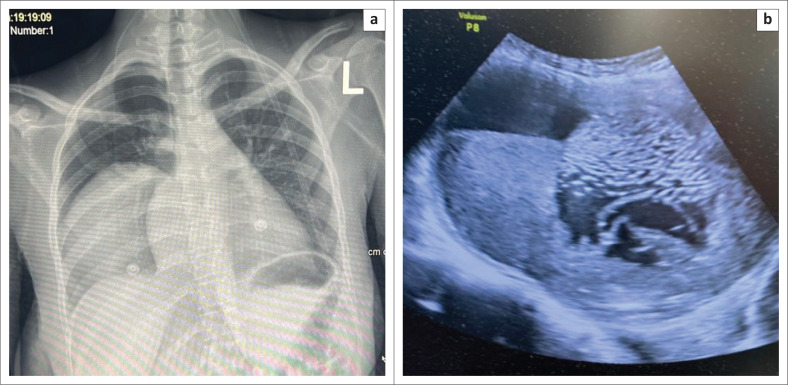
(a) Chest X-ray displaying right sided pulmonary cystic lesion. (b) Sagittal ultrasound view of the uterus demonstrating a cystic lesion with septation and a daughter cyst.

Initial investigations included full blood count, urea and electrolytes, C-reactive protein, procalcitonin, sputum for Gene Xpert and coronavirus disease (COVID) polymerase chain reaction. Imaging included a chest X-ray and pelvic ultrasound. The full blood count showed a microcytic, hypochromic anaemia with Hb 9.6 g/dL and mean corpuscular volume (MCV) of 94.4 fL. Septic markers were not elevated (Table 1). Blood cultures were negative after 5 days. Gene Xpert and severe acute respiratory syndrome coronavirus 2 (SARS- CoV-2) tests were negative (Table 1). Chest X-ray showed a large right-sided pleural opacity, consistent with a simple effusion involving the lower and middle lobes (Figure 1). Pelvic ultrasound revealed multiple intrauterine cystic lesions and solid areas, suggestive of retained products of conception (Figure 1). Because of diagnostic uncertainty, the patient was prepared for uterine evacuation with consent for possible hysterectomy. Intraoperatively, 1000 mL of purulent, viscous fluid containing membranes and cystic structures was evacuated, and concern for uterine rupture led to an exploratory laparotomy.

**TABLE 1:** Haematology, microbiology and pathology results.

Item	Results	Reference range
White cell count (10^9^/L)	13.21	3.92–10.40
Red cell count (10^12^/L)	3.22	4.19–5.85
Haemoglobin (g/dL)	9.6	13.4–17.5
MCV (fL)	94.4	83.1–100.6
Platelet count (10^9^/L)	444	171–388
Sodium (mmol/L)	137	136–145
Potassium (mmol/L)	5	3.5–5.1
Chloride (mmol/L)	101	98–107
Urea (mmol/L)	1.3	2.1–7.1
Creatinine (mmol/L)	43	64–104
eGFR (mL/min/1.72m^2^)†	> 60	> 60
C-reactive protein (mg/L)	3	< 10
Procalcitonin (μg/L)	1.10	> 0.5
D-dimer (mg/L)	1.02	0.00–0.25
Sputum analysis	GXP: Neg	-
Pleural tap (cells/μL)	Cell count: 0 Lymphocytes: 0 Erythrocytes: 0	- - -
**Cystic fluid content:**		
Auramine stain	Negative	-
GeneXpert, MTB PCR	Negative	-
Microscopy, culture and sensitivity	Neutrophils: Not observed Lymphocytes: Not observed Epithelial cells: Moderate (2+) Organisms: No bacteria observed Bacterial culture: No growth after 2 days No anaerobic growth after 2 days incubation	- - - - - -
Blood cultures	No growth after 5 days	-

† , MDRD formula.

MCV, mean corpuscular volume; eGFR, estimated glomerular filtration rate; MDRD, modification of diet in renal disease; MTB PCR, mycobacterium tuberculosis polymerase chain reaction; GXP, GeneXpert; Neg, negative.

**FIGURE 2:**
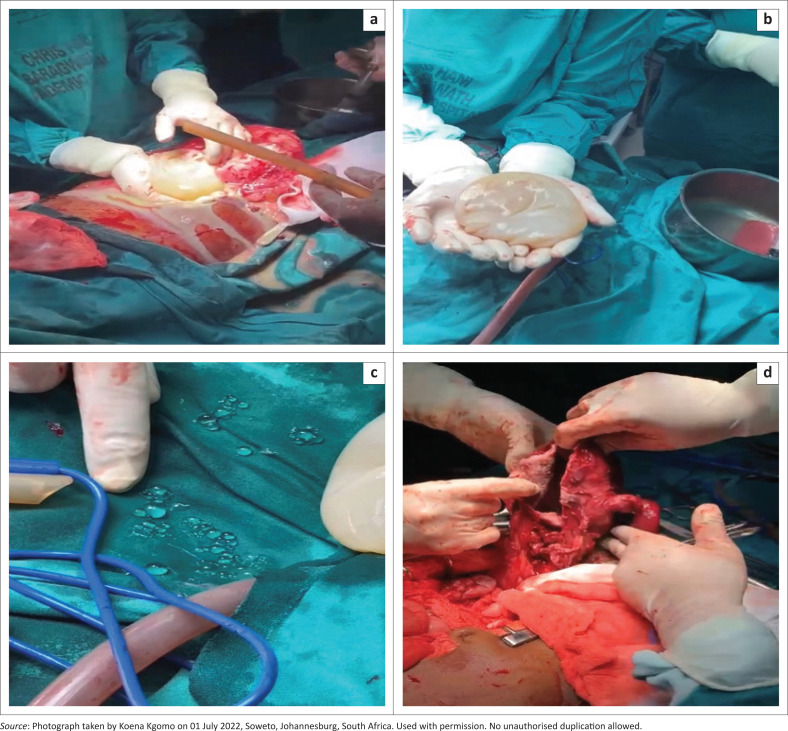
(a) Ruptured cystic mass found during exploratory laparotomy. (b) Hydatid cyst after surgical excision. (c) Daughter cysts found within the uterine and cyst cavity. (d) Thin walled, grossly distended uterus post excision (enucleation) of the cystic mass.

Surgical findings included a pale, non-blanching, thin-walled uterus distended to 22 cm, with a posterior wall rupture. The uterine cavity contained a large ruptured cystic mass with multiple daughter cysts. Both fallopian tubes were grossly swollen and distorted (Figure 2). The ovaries appeared unaffected. Dense adhesions between the uterus and bowel posteriorly and omentum anteriorly were noticed. A total abdominal hysterectomy and bilateral salpingectomy were performed. After the procedure, the abdomen and pelvis were extensively washed out using 5 L of sterile water. No other intra-abdominal cysts were identified.

Histopathology showed a cyst wall composed of an acellular laminated membrane without dysplasia or malignancy. No granulomas were present. Features were consistent with a hydatid cyst. Uterine tissue displayed chronic endometritis and signs of previous placental implantation. The fallopian tubes showed moderate chronic active salpingitis. Ziehl-Neelsen stain and Alcian Blue and Periodic Acid-Schiff stain were negative for acid-fast bacilli and fungi, respectively.

**FIGURE 3:**
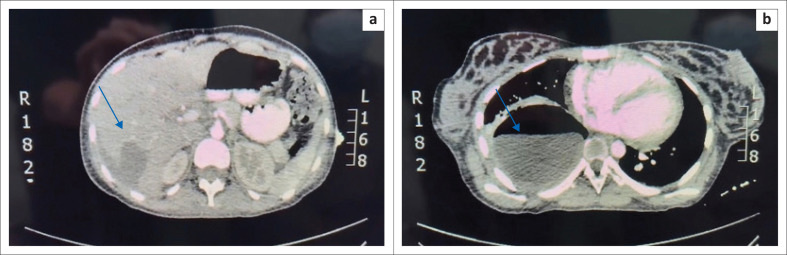
(a) Subcapsular fluid collection at T6-10. (b) Right lower lobe with large cyst consistent with a ruptured hepatic hydatid cyst at level T11-L2.

Postoperative computerised tomography (CT) imaging of the chest and abdomen revealed a large cystic pulmonary lesion in the right lower lobe, characteristic of pulmonary hydatid disease, along with a subcapsular fluid collection, consistent with a ruptured hepatic hydatid cyst. All other abdominal organs showed no abnormalities (Figure 3).

The patient was referred to internal medicine and infectious disease specialists. She was started on oral albendazole 400 mg 12 hourly for 6 weeks. Surgical management of the lung cyst via thoracotomy and enucleation was advised by the cardiothoracic unit. However, the patient declined further treatment and discharged herself against medical advice. She returned to the Eastern Cape and has since been lost to follow-up.

## Discussion

Hydatid disease affects 2–3 million individuals globally involving the liver and lungs in over 80% of cases.^3,6^ Pelvic involvement is rare, comprising only about 2% of cases, with the uterus among the least common sites. Involvement of the female genital tract is often attributed to its rich vascular supply.^7^

Clinical manifestations of uterine hydatid cysts are non-specific – abdominal pain, weight loss, pressure symptoms, menstrual disturbances and infertility may occur.^3^ In pregnancy, diagnosis is especially challenging because of overlapping features with obstetric complications and limited imaging options.^8^

Diagnosis is based on imaging studies and serological tests. Ultrasound is the preferred modality, with a sensitivity of 93% – 98%. To guide treatment decisions, the World Health Organization (WHO) developed a classification system categorising cystic echinococcus (CE) ranging from CE1, representing active cysts, to CE5, representing inactive cysts, to guide treatment decisions.^7^ Enzyme-linked immunosorbent assay (ELISA) detects IgG antibodies against hydatid cyst antigens and has high sensitivity (95%) and specificity (94%). Indirect haemagglutination, which has specificity of 87.5%, can also be used.^7^

Echinococcosis is recognised by the WHO as one of over 20 neglected tropical diseases, with calls for global control efforts.^9^ Management options include pharmacological therapy, percutaneous interventions, surgery and watchful waiting. Choice of therapy depends on cyst type, location, complications and local expertise.^10^ Referral to specialised centres may be advisable to ensure improved ongoing patient management and care.^1^

Albendazole, administered at 10 mg/kg/day – 15 mg/kg/day in divided doses, is the drug of choice for treatment of CE. Treatment may extend up to 6 months. Albendazole is often used preoperatively to reduce cyst viability or postoperatively to minimise recurrence risk. However, its use during pregnancy is avoided, particularly in the first trimester, because of the potential teratogenic effects. Deworming during pregnancy requires careful risk–benefit assessment and is typically deferred unless the clinical situation necessitates intervention.^11^ The WHO Informal Working Group on Echinococcosis (IWGE) advocates a stage-specific approach.^2^

Surgical resection remains the primary treatment approach for large or complicated cysts causing pressure effects. Percutaneous techniques, involving aspirating the cyst contents under image guidance and injecting a scolicidal agent to kill the parasite, are suitable for selected patients, especially with hepatic involvement. Because of limited experience with uterine hydatid disease, management strategies must be individualised. Recognised treatment centres are preferred for managing complex CE cases.^1^

Because echinococcosis rarely affects the uterus, it is often not considered in the differential diagnosis of pelvic cysts. This case illustrates the need for heightened clinical suspicion in endemic regions or among individuals with a history of livestock and dog exposure. The timing of infection in this patient is uncertain but likely occurred in early pregnancy, a naturally immunosuppressed state.^12^ This immunocompromise may have contributed to disseminated disease, preterm delivery and foetal growth restriction. Whether the uterine rupture preceded presentation or resulted from evacuation remains unclear because of the friable, thin-walled uterus. Nonetheless, earlier pregnancy imaging might have enabled timely diagnosis and altered the clinical outcome.

## Conclusion

This case represents an exceptionally rare occurrence of uterine echinococcosis in South Africa, presenting in the postpartum period. It underscores the importance of considering hydatid disease as a differential for pelvic cystic lesions, particularly in patients from endemic areas. Timely diagnosis through early recognition, comprehensive multidisciplinary care and appropriate imaging is essential to improving patient outcomes. However, surveillance alone is insufficient – robust prevention strategies must be prioritised, particularly in high-risk settings. To effectively reduce the burden of this disease, integrated efforts focused on awareness, prevention and vigilant surveillance are necessary.
